# Physalin H from *Solanum nigrum* as an Hh signaling inhibitor blocks GLI1–DNA-complex formation

**DOI:** 10.3762/bjoc.10.10

**Published:** 2014-01-13

**Authors:** Midori A Arai, Kyoko Uchida, Samir K Sadhu, Firoj Ahmed, Masami Ishibashi

**Affiliations:** 1 Graduate School of Pharmaceutical Sciences, Chiba University, 1-8-1 Inohana, Chuo-ku, Chiba 260-8675, Japan; 2 Pharmacy Discipline, Life Science School, Khulna University, Khulna-9208, Bangladesh; 3 Department of Pharmaceutical Chemistry, University of Dhaka, Dhaka-1000, Bangladesh

**Keywords:** Hedgehog inhibitor, Hedgehog signal, natural products, physalins, *Solanum nigrum*

## Abstract

Hedgehog (Hh) signaling plays an important role in embryonic development, cell maintenance and cell proliferation. Moreover, Hh signaling contributes to the growth of cancer cells. Physalins are highly oxidized natural products with a complex structure. Physalins (**1**–**7**) were isolated from *Solanum nigrum* (Solanaceae) collected in Bangladesh by using our cell-based assay. The isolated physalins included the previously reported Hh inhibitors **5** and **6**. Compounds **1** and **4** showed strong inhibition of GLI1 transcriptional activity, and exhibited cytotoxicity against cancer cell lines with an aberrant activation of Hh signaling. Compound **1** inhibited the production of the Hh-related proteins patched (PTCH) and BCL2. Analysis of the structures of different physalins showed that the left part of the physalins was important for Hh inhibitory activity. Interestingly, physalin H (**1**) disrupted GLI1 binding to its DNA binding domain, while the weak inhibitor physalin G (**2**) did not show inhibition of GLI1-DNA complex formation.

## Introduction

Hedgehog (Hh) signaling plays an important role in embryonic development and adult tissue maintenance [1–2]. Once the Hh protein binds and inhibits its receptor patched (PTCH), Smoothened (SMO) makes GLIs, which are zinc-finger transcription factors, free to move in the nucleus from the GLIs–SuFu complex. The GLIs then bind to their consensus DNA sequence and turn on the expression of the target gene [3–4]. Although this signal has been identified as one of the key intercellular signals in many fundamental processes, aberrant activation of this signaling pathway plays a crucial role in the development of tumors such as basal-cell carcinomas and small-cell lung cancer [5–7]. To find a drug for the treatment of cancer, Hh inhibitors have been recently developed. SMO inhibitors are a major category of Hh signaling inhibitors (cyclopamine [8–9], Cur-61414 [10] and SANTs [11]). Robotnikinin interacts with the Shh protein [12], and JK184 induces Hh inhibition through class IV alcohol dehydrogenase [13]. GANT-61 is an inhibitor, which disturbs GLIs binding to their binding site (GACCACCCA) in the promoter region of the target genes [14]. The AAA+ ATPase motor cytoplasmic dynein has been found to be the molecular target for Hh pathway inhibitors (HPIs) [15–16].

**Figure 1:**
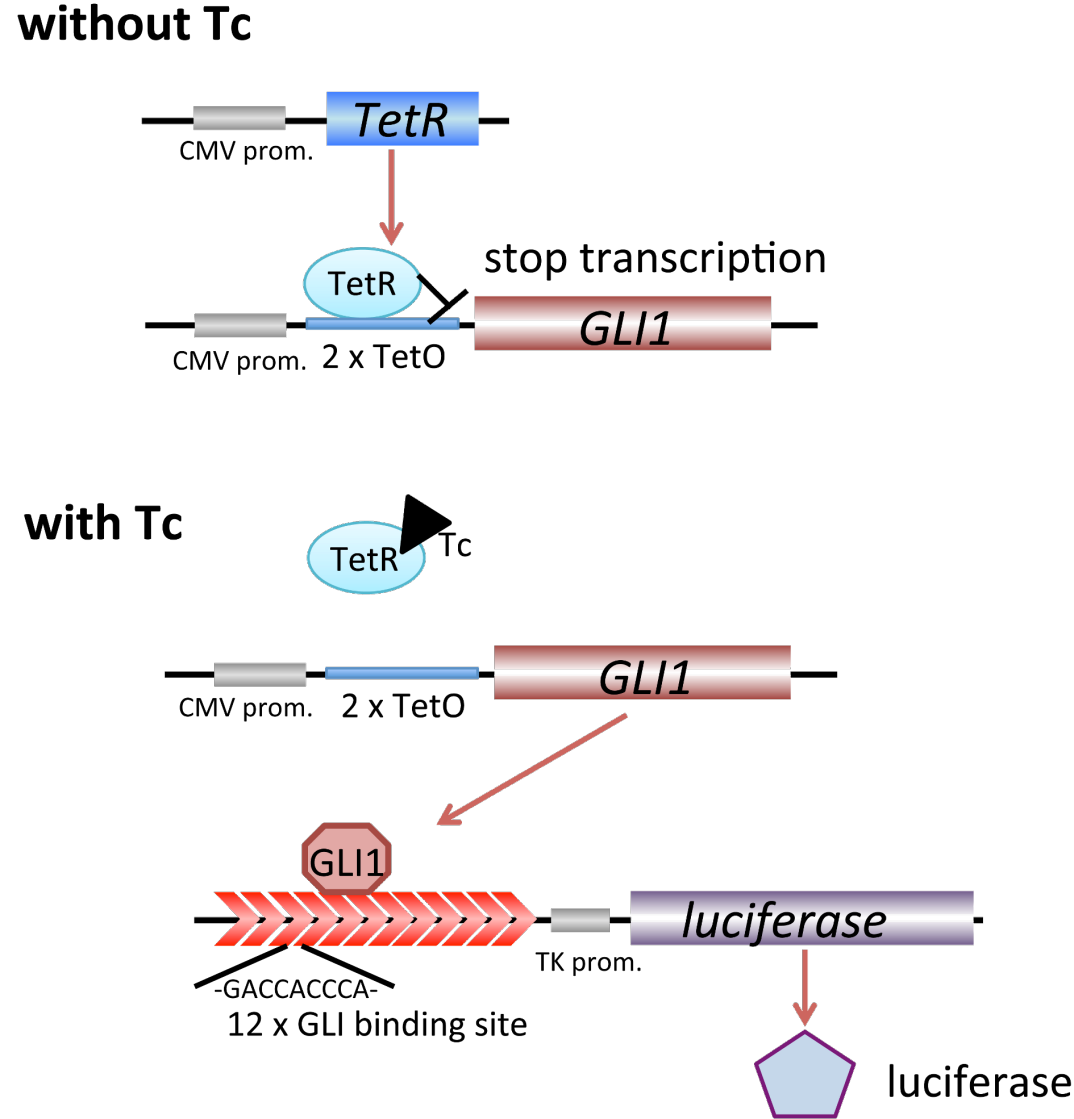
Schematic procedure of the constructed assay system; Tc (tetracycline), TetR (tetracycline repressor), TetO (tetracycline operator).

Previously, we have constructed a cell-based assay involving a tetracycline-regulated (T-Rex) system for Hh/GLI-mediated transcriptional inhibitors (Figure 1) [17–22]. Naturally occurring Hh inhibitors such as physalin F (**5**; IC_50_, 0.66 μM) [17], physalin B (**6**; IC_50_, 0.62 μM) [17], colubrinic acid (IC_50_, 38 μM) [18], caldenolides (IC_50_, 0.1–0.45 μM) [19], taepeenin D (IC_50_, 1.6 μM) [20] and a flavonoid glycoside (IC_50_, 0.5 μM) [21] were isolated by using this screening assay. Recently, we found that vitetrifolin D (IC_50_, 20.2 μM) is the first naturally occurring Hh inhibitor that impairs GLI1–DNA binding [22]. Because of the mutation of components of the Hh signal such as SMO, more downstream events such as the binding of GLI1 on DNA would be affected by the inhibition because of the mutation of components in many cancers. In this study, we report the isolation of four physalins (**1**–**4**) along with the previously reported Hh inhibitors **5** and **6**. In addition, we evaluated the Hh inhibitory activity by assessing the cytotoxicity against cancer cells and the expression of Hh-related proteins. Physalin H (**1**) inhibited GLI1 binding to its DNA binding domain as assessed by our previously reported electrophoresis mobility shift assay (EMSA).

## Results and Discussion

### Isolation of physalins

**Figure 2:**
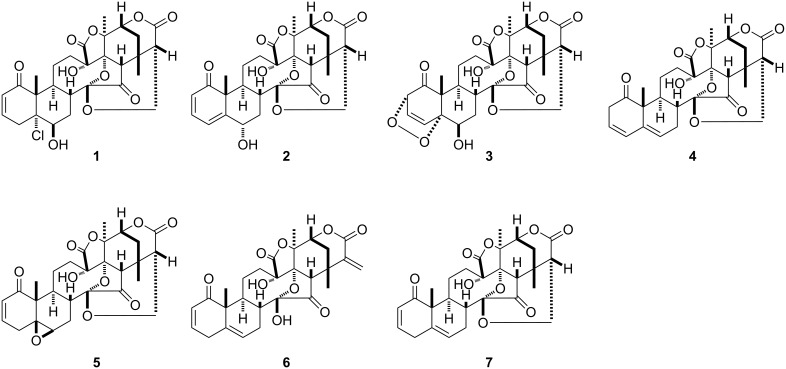
Isolated compounds from *Solanum nigrum*.

Among our extract library, *Solanum nigrum* was identified as an active plant with our cell-based assay screening. After the removal of chlorophyll from the MeOH extract of *Solanum nigrum* (leaves) (7.9 g) by Diaion HP-20, each MeOH soluble part of fraction 1A (3.7 g) and fraction 1B (1.2 g) were suspended in H_2_O/MeOH 9:1. The suspension thus obtained was successively partitioned between hexane, EtOAc and *n*-BuOH. Consecutive steps of silica gel column chromatography, and reversed-phase high-performance liquid chromatography (HPLC) of the active EtOAc extracts of fraction 1A and 1B yielded physalin H (**1**) [23], physalin G (**2**) [24], physalin K (**3**) [25], isophysalin B (**4**) [26], physalin F (**5**) [27] and a mixture of physalin B (**6**) [28] and physalin C (**7**) [29]. The structures of these compounds were identified by comparing their spectral data with those reported in the literature (Figure 2).

### Evaluation of Hh inhibitory activity of isolated physalins

**Figure 3:**
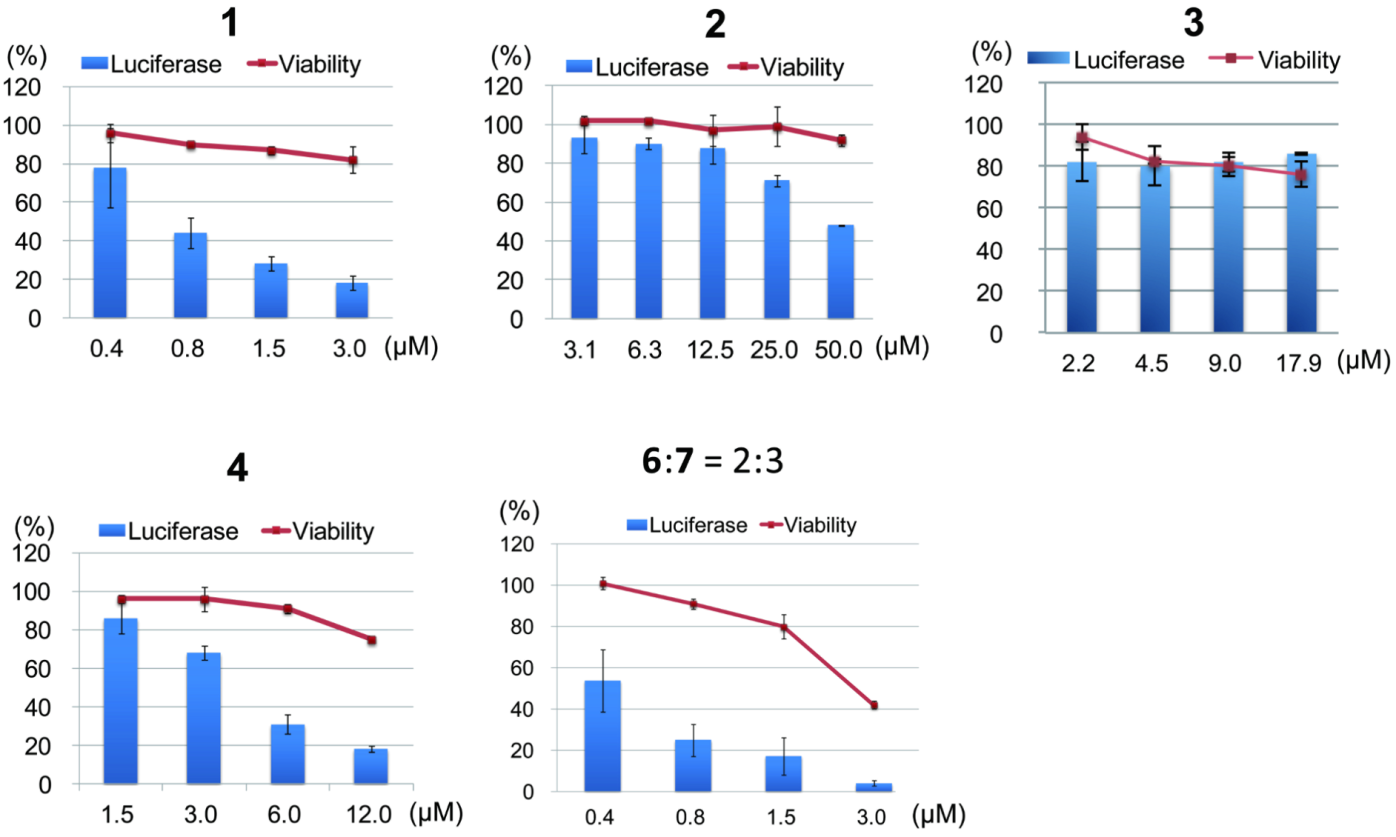
GLI1 transcriptional activity and cytotoxicity. Reporter activity (solid columns) and cell viability (solid curves) of compouds **1**–**4** and mixture of **6** and **7** (2:3). The assays were performed at 0.05% DMSO (*n* = 3). Error bars represent SD.

**Figure 4:**
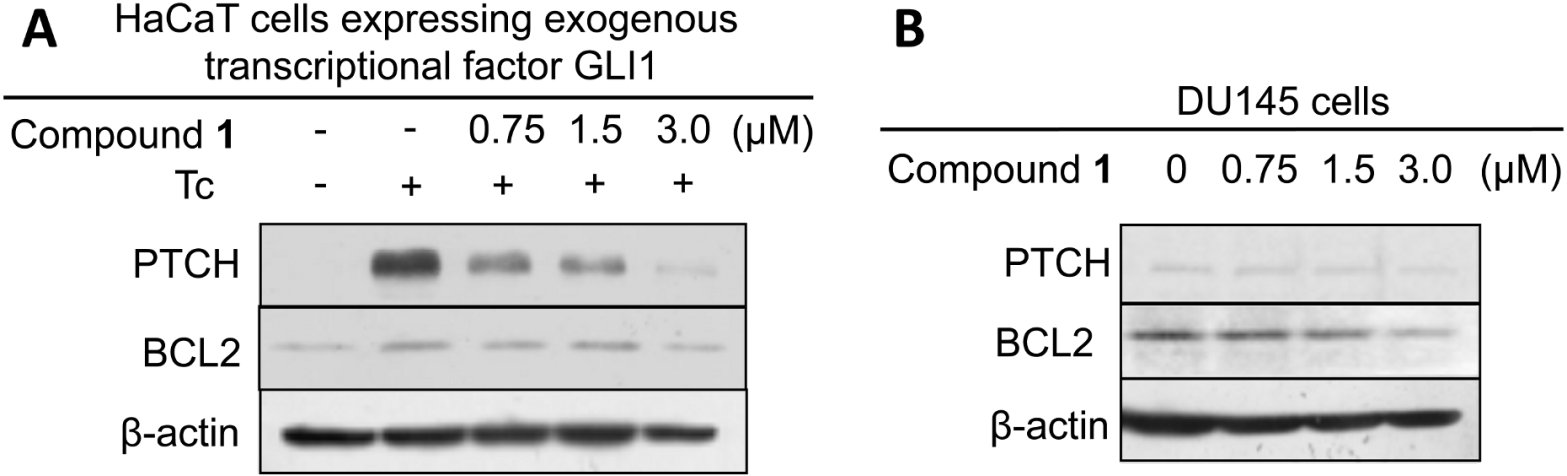
Inhibition of protein expression by compound **1**. (A) Western blot analysis of PTCH, BCL-2 protein levels in GLI1-overexpressing HaCaT cells after treatment with **1**. (B) Western blot analysis of PTCH, BCL-2 protein levels in DU145 cells after treatment with **1**.

We evaluated the Hh inhibitory activity of the isolated compounds by using a previously described method [18]. Detection of the Hh inhibitory activity was based on the decrease in the luminescence value due to reduced luciferase expression by GLI1 compared to that of the control (DMSO). In addition we always checked the cytotoxicity of the compounds against the assay cells, because a decrease in the number of living cells also correlates with a reduced luciferase activity. Physalin H (**1**) and isophysalin B (**4**) showed a dose-dependent inhibition of luciferase production (IC_50_ were 0.70 and 4.2 μM, respectively) with a low cytotoxicity against assay cells (Figure 3). Physalin G (**2**) showed a weak Hh inhibition (IC_50_, 47.1 μM). Physalin K (**3**) did not show Hh inhibition at all, even at a concentration of 17.9 μM. Previously, we reported on the isolation and evaluation of Hh inhibitory activities of physalin F (**5**) (IC_50_, 0.66 μM) and physalin B (**6**) (IC_50_, 0.62 μM) [17]. In this study, physalin B (**6**) was isolated as a mixture with physalin C (**7**) at a ratio of 3:2. An equilibrium between **6** and **7** might exist under weakly acidic conditions, because the ratio of the mixture changed from 3:2 to 1:1 in a deuterated chloroform (CDCl_3_) solution after two months. Further, we examined the cytotoxicity against cancer cells, human pancreatic (PANC1) and prostate (DU145) cancer cell lines, in which Hh signaling is aberrantly activated (Table 1). Physalin H (**1**) (IC_50_, 0.70 μM) showed cytotoxicity against PANC1 (IC_50_, 5.7 μM) and DU145 (IC_50_, 6.8 μM). In addition, isophysalin B (**4**) and previously reported **5** and **6** also exhibited strong cytotoxicity against PANC1 (IC_50_ were 12.0, 2.7 and 5.3 μM, respectively). A moderate selectivity between differentiating cancer cells and normal cells was observed for active compounds **1**, **4**, **5** and **6**, because the survival of C3H10T1/2 cells was not significantly affected by a treatment with these compounds. C3H10T1/2 is a normal cell line in which the Hh signalling pathway is active but does not contribute to abnormal cell survival. Interestingly, the right parts of these isolated physalins, except **7**, were identical. Therefore, the difference in Hh inhibitory activity can be attributed to the left part of their structures. The difference in the Hh inhibitory activity between **4** (IC_50_, 4.2 μM) and **6** (IC_50_, 0.62 μM) indicates that an α,β-unsaturated ketone unit present in the physalin structure may be a good nucleophile acceptor and contribute to the Hh inhibition. In addition, a conjugated dienone system in **2** and a peroxy bridge in **3** may be associated with decreased Hh inhibitory activity. Western blot analyses were performed with physalin H (**1**), which was the most active compound, except previously reported compounds **5** and **6**. Physalin H (**1**) showed an inhibition of Hh- related protein expression (Figure 4). The effect of **1** on the expression of a Hh membrane receptor, PTCH, and an anti-apoptosis protein BCL2 in HaCaT cells expressing exogenous GLI1 (reporter assay cells) is shown in Figure 4A. At a concentration of 3.0 μM, physalin H (**1**) inhibited the expression of both proteins. In addition, at a concentration of 3.0 μM, physalin H (**1**) decreased the expression of BCL2 in DU145 cancer cells, in which Hh signaling was activated (Figure 4B). However, the expression of PTCH was only slightly affected.

**Table 1:** GLI1 transcriptional activity and cytotoxicity.

Compound	GLI1 transcriptional inhibition, IC_50_ (μM)	Cytotoxicity, IC_50_ (μM)		
		PANC1	DU145	C3H10T1/2

physalin H (**1**)	0.70	5.7	6.8	>6.0
physalin G (**2**)	47.1	–	>12.0	–
physalin K (**3**)	–	–	–	–
isophysalin B (**4**)	4.2	12.0	>12.0	>12.0
physalin F (**5**)^a^	0.66^a^	2.7^a^	–	15.0^a^
physalin B (**6**)^a^	0.62^a^	5.3^a^	–	17.0^a^
**6** and **7** mixture (2:3)	0.40	4.0	3.6	>6.0

^a^Data from [17].

### Inhibition of GLI1–DNA complex formation by physalin H (**1**)

**Figure 5:**
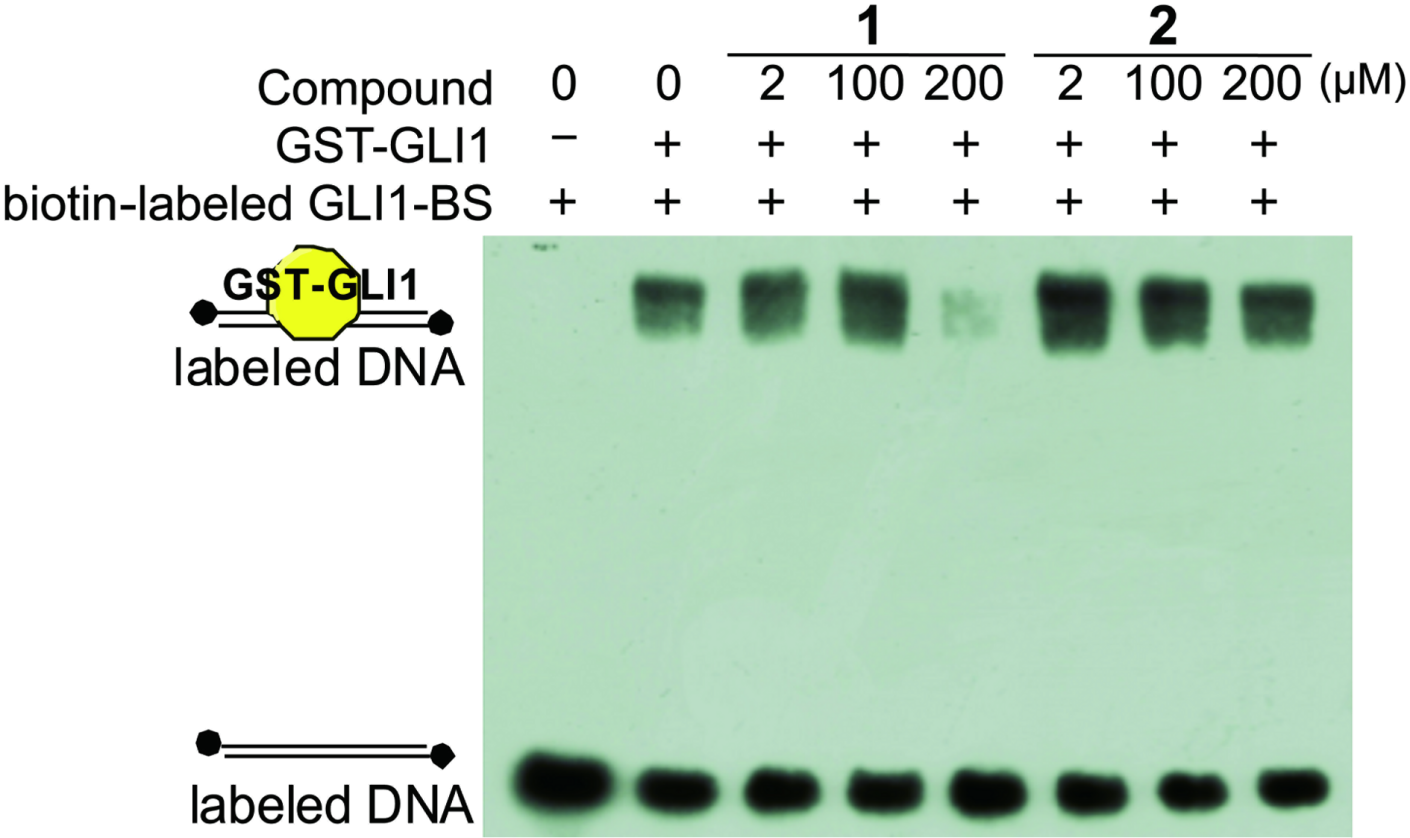
Inhibitory activity of GLI1–DNA-complex formation by electron mobility shift assay (EMSA). GST–GLI1 (171-515aa), DNA containing the GLI1 binding site (GLI1–BS); 5’-AGCTACCTGGGTGGTCTCTTCGA-3’. The reproducibility was checked in five individual experiments.

Disruption of the GLI1–DNA complex in the final stage of this signaling pathway is one of the most attractive molecular mechanisms of Hh inhibitors. Recently, we reported the first small molecule, which directly inhibits the GLI1–DNA complex formation [22].GANT61, a synthetic compound, has been reported to inhibit the GLI1–DNA-complex formation after cells were treated with this compound. The author speculated that GANT61 might affect modifications of the GLI1 protein expression, which causes disruption of the GLI1–DNA complex. In our previous study [22], we constructed an electrophoretic mobility shift assay (EMSA) for the detection of the GLI1–DNA complex. The assay uses a slightly modified biotin-tagged DNA and modified amino acids sequences of GLI1 compared to those reported in literature [30–32]. In a similar manner as described in [22], GLI1 protein was expressed in *Escherichia coli* as a 171–515 amino chain, including the five Zn finger regions. Horseradish peroxidase (HRP)-conjugated streptavidin detected free “biotin-labeled GLI1-BS” (DNA containing GLI1 binding site; biotin-AGCTACCTGGGTGGTCTCTTCGA; the underlined 9 bps are a consensus sequence [30]; Figure 5, lane 1). After mixing with GST–GLl1, GLI1–BS and GST–GLl1 the complex was detected in the upper line, because of an increasing molecular weight (low mobility; lane 2). By using this EMSA, the inhibitory activity of physalin H (**1**, IC_50_; 0.70 μM) and inactive physalin G (**2**, IC_50_; 47.1 μM) was examined. Under these experimental conditions, physalin H (**1**) clearly inhibited the formation of GLI1–DNA complex at 200 μM. On the other hand, physalin G (**2**), which is similar in the right part of its structure to physalin H (**1**), did not inhibit the complex formation even at 200 μM. These results suggested that one of the Hh inhibitory mechanisms of physalin H (**1**) involves inhibition of GLI1–DNA-complex formation.

## Conclusion

We isolated seven physalins from *S. nigrum* by using Hh inhibitory activity-guided isolation. Among them, four compounds (**1**, **4**, **5** and **6**) showed dose-dependent GLI1-transcriptional inhibitory activity. In addition, these physalins were found to be cytotoxic to cancer cells with an aberrant Hh signaling pathway and inhibited the expression of Hh signaling-related proteins. Structure–activity relationships were observed in the Hh inhibitory activity of physalins and the left part of the structure was found to have an important role in Hh inhibition. Moreover, physalin H (**1**) inhibited the formation of GLI1–DNA complex, but inactive physalin G (**2**) did not. To our knowledge, this is the second study in which the direct inhibition of the formation of the GL1–DNA complex by Hh inhibitors has been reported.

## Experimental

### General experimental procedures

The NMR measurements were carried out with JEOL ECP400 and ECP600 spectrometers in deuterated solvents and the residual proton chemical shift was taken as an internal standard. MS data were obtained with a JEOL JMS-T100LP (ESI). IR spectra were measured by using the ATR (attenuated total reflection) method on a Jasco FT-IR 230 spectrophotometer. The DNA concentration was measured by a NanoDrop 2000 (Thermo Fisher Scientific), and the protein concentration was measured by an UVmini 1240 UV–vis Spectrophotometer (SHIMADZU, Kyoto, Japan). The procedures for the GLI1-mediated transcriptional activity assay, Western blot analysis and cytotoxicity test were as previously described [18]. The GST–GLI1 protein was produced as previously described [22].

#### Plant material

The plant *Solanum nigrum* was collected in Bangladesh in 2011. A voucher specimen (KKB204) was deposited in the Ishibashi laboratory at the Chiba University.

#### Extraction and isolation

After the removal of chlorophyll from a MeOH extract of *S. nigrum* (leave) (7.9 g) by Diaion HP-20, fraction 1A (3.7 g) was then partitioned between hexane, EtOAc and *n*-BuOH. The active EtOAc extract (623.4 mg) was subjected to silica gel column chromatography (30 × 180 mm; CHCl_3_/MeOH 1:0 to 0:1, 0:1 + 0.1% TFA) to give 7 fractions (15A–15G). Fraction 15C (68.5 mg) was subjected to ODS HPLC (COSMOSIL Packed Column 5-CN-MS 10 × 250 mm; MeCN/H_2_O 68:35, flow rate 2.0 mL/min, UV detection at 254 nm) to give compound **2** (10.1 mg, *t*_R_ 10 min), compound **3** (2.4 mg, *t*_R_ 17 min) and compound **1** (3.8 mg, *t*_R_ 25 min). Fraction 1B (1.2 g) was partitioned between hexane, EtOAc and *n*-BuOH. The active EtOAc extract (294.5 mg) was subjected to silica gel column chromatography (25 × 200 mm; CHCl_3_/MeOH 40:1 to 0:1, 0:1 + 0.1% TFA) to give 6 fractions (4A–4F). Fraction 4A (179.6 mg) was subjected to silicagel column chromatography (20 × 200 mm; hexane/EtOAc 2:1 to 0:1, MeOH) to give 5 fractions (5A–5E). Fraction 5C (50.7 mg) was subjected to ODS HPLC (COSMOSIL Packed Column 5-CN-MS 10 × 250 mm; MeCN/H_2_O 2:3, flow rate 2.0 mL/min, UV detection at 254 nm) to give 8 fractions (6A–6H). Fraction 6E (20.0 mg) was subjected to ODS HPLC (Develosil Packed Column ODS-HG-5, 10 × 250 mm; MeCN/H_2_O 2:3, flow rate 2.0 mL/min, UV detection at 254 nm) to give compound **5** (7.0 mg, *t*_R_ 31 min). Fraction 5B (91.0 mg) was subjected to ODS HPLC (COSMOSIL Packed Column 5-CN-MS, 10 × 250 mm; MeCN/H_2_O 2:3, flow rate 2.0 mL/min, UV detection at 254 nm) to give 6 fractions (7A–7F). Fraction 7E (11.4 mg) was subjected to ODS HPLC (Develosil Packed Column ODS-HG-5, 10 × 250 mm; MeCN/H_2_O 1:1, flow rate 2.0 mL/min, UV detection at 254 nm) to give compound **4** (4.0 mg, *t*_R_ 32 min). Fraction 7D (34.0 mg) was subjected to ODS HPLC (Develosil Packed Column ODS-HG-5, 10 × 250 mm; MeCN/H_2_O 2:3, flow rate 2.0 mL/min, UV detection at 254 nm) to give a 2:3 mixture of compounds **6** and **7** (2.0 mg, *t*_R_ 10 min) and compound **4** (1.7 mg, *t*_R_ 24 min). Fraction 4B was subjected to ODS HPLC (COSMOSIL Packed Column 5-CN-MS, 10 × 250 mm; MeCN/H_2_O 37:63, flow rate 2.0 mL/min, UV detection at 254 nm) to give compound **3** (1.7 mg, *t*_R_ 24 min) and compound **1** (3.3 mg, *t*_R_ 30 min).

#### Electrophoretic mobility shift assay (EMSA)

The double-stranded (ds) DNA fragments containing a GLI1 binding site (GLI1–BS) were prepared by annealing of single strand (ss) DNA; for biotin-labeled GLI1–BS, 5’-biotin-AGCTACCTGGGTGGTCTCTTCGA-3’ and 5’-biotin-TCGAAGAGACCACCCAGGTAGCT-3’. 10 μL of the ssDNA (100 pmol/μL in TE buffer (NIPPON GENE, Tokyo, Japan)) were mixed and annealed by PCR Thermal Cycler Dice (Takara, Kyoto, Japan) (95 °C, 30 s; 72 °C, 2 min; 37 °C, 2 min; 25 °C, 2 min). For EMSA experiments, dsDNA TE buffer solutions were prepared as follows: biotin-labeled GLI1–BS (50 fmol/μL), unlabeled mutGLI1–BS and unlabeled GLI1–BS (5 pmol/μL). These solutions were stored below −20 °C.

The assays were performed with a Light Shift Chemiluminescent EMSA Kit (Thermo). The binding reaction was set up as follows: 70 ng of GST–GLI1 protein, biotin-labeled GLI1–BS (20 fmol), and compound in DMSO (final DMSO concentration was 0.6%) were combined in 2 μL of 10 × binding buffer (kit), (5 mM MgCl_2_, 4% glycerol) on ice (total volume 20 μL). After incubation for 20 min on ice, protein–DNA complex, free DNA and free protein were separated on a 6% native polyacrylamide gel containing Tris-Borate-EDTA (TBE; 45 mM tris-borate, 1.0 mM EDTA). After transfer of DNA onto a Biodyne B nylon membrane 0.45 µm (Pall Gelman Laboratory, Ann Arbor, MI) and DNA crosslinking by UV transilluminator (312 nm, 15 min), biotin-labeled DNA was detected by HRP-conjugated streptavidin, following the manufactures’ protocols.
